# Supplementary material for: A STAT3-based gene signature stratifies glioma patients for targeted therapy
Source: Nat Commun. 2019 Aug 9;10:3601. doi: 10.1038/s41467-019-11614-x (PMC6689009; doi:10.1038/s41467-019-11614-x)
Supplement: Supplementary file 1 — Supplementary Information [file 41467_2019_11614_MOESM1_ESM.pdf]

Supplementary Information

A STAT3 based gene signature stratifies glioma patients for targeted therapy

Tan *et al*.

Supplementary Figures

Supplementary Figure 1. *STAT3* knockdown reduced viability, gliomasphere-forming ability and clonogenicity.

Supplementary Figure 2. NNI-*STAT3* signature validation in TCGA database.

Supplementary Figure 3. NNI patient tumors are IDH-wild-type with variable *STAT3* expression and are stratified by *STAT3* functionally-tuned gene signature.

Supplementary Figure 4. Mechanistic gene candidates identified by NNI-*STAT3* gene signature representing chemoresistance to STAT3 inhibitor.

Supplementary Figure 5. Sensitization of *STAT3*-low glioma cells and immunohistochemical (IHC) staining and quantification of pSTAT3 and IGF-1R in patient-derived xenograft (PDX) tumors.

Supplementary Figure 6. Proposed mechanism in *STAT3*-low (chemoresistant) GBM cells.

Supplementary Figure 7. IC_{50} values of GBM-propagating cells (GPCs).

Supplementary Figure 8. Validation of NNI tumors and GPCs with international collections.

Supplementary Figure 9. Response to NT157, a selective inhibitor targeting IGF-1R and STAT3 signaling pathways.

Supplementary Tables

Supplementary Table 1. (a) Contingency tables, (b) Univariate and multivariate analyses.

Supplementary Table 2. Kaplan-Meier statistics.

Supplementary Table 3. List of protein tyrosine kinases up/down-regulated in *STAT3*-high and –low GPCs upon treatment with AZD1480.

Supplementary Table 4. List of top synergistic compounds able to reverse *STAT3*-high GBM disease signature (temozolomide as reference compound).

Supplementary Data

Supplementary Data 1. List of genes comprising the *STAT3* functionally-tuned gene signature.

Supplementary Data 2. Gene Set Enrichment Analysis (GSEA) ranked gene list.

Supplementary Data 3. Winnowed list of genes contributing to chemoresistance.

Supplementary References

Supplementary Figures

Supp Figure 1

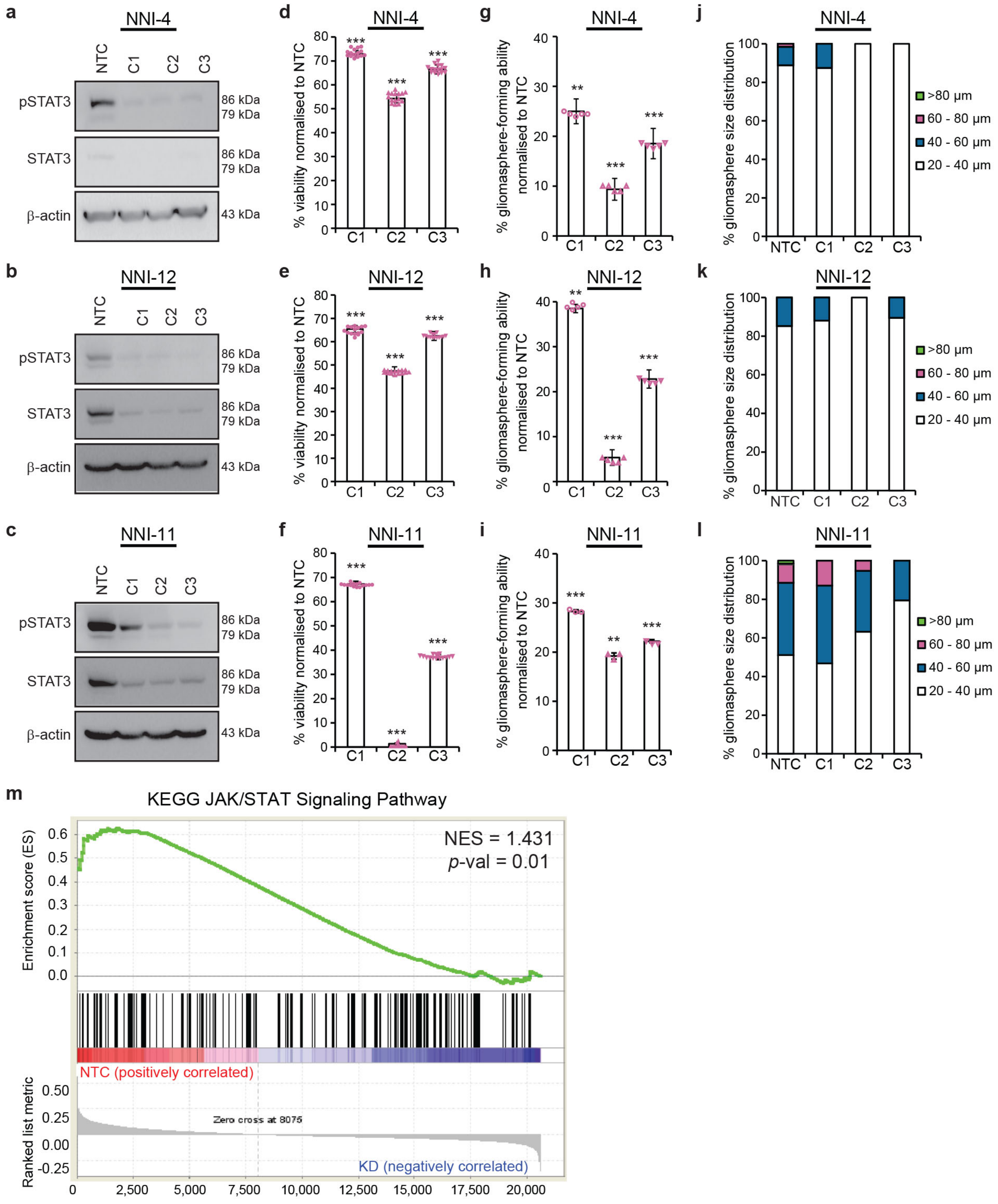

Supplementary Figure 1 *STAT3* knockdown reduced viability, gliomasphere-forming ability and clonogenicity. NNI-4, NNI-11 and NNI-12 were subjected to lentiviral-mediated *STAT3* knockdown (NTC, non-targeting control; 3 sh*STAT3* knockdown clones, C1, C2 and C3). The knockdown clones were verified by **a-c** immunoblot analysis; **d-f** cell viability assay; **g-i** clonogenicity, and **j-l** gliomasphere size distribution. **, p<0.01; ***, p<0.001; versus NTC. For statistical analysis, two-sided Student's t test was used, Error bars represent standard deviation of the mean. **m** Gene Set Enrichment Analysis (GSEA) in *STAT3* knockdown expression profile revealed down-regulation of the JAK-STAT signaling pathway.

Supp Fig. 2.

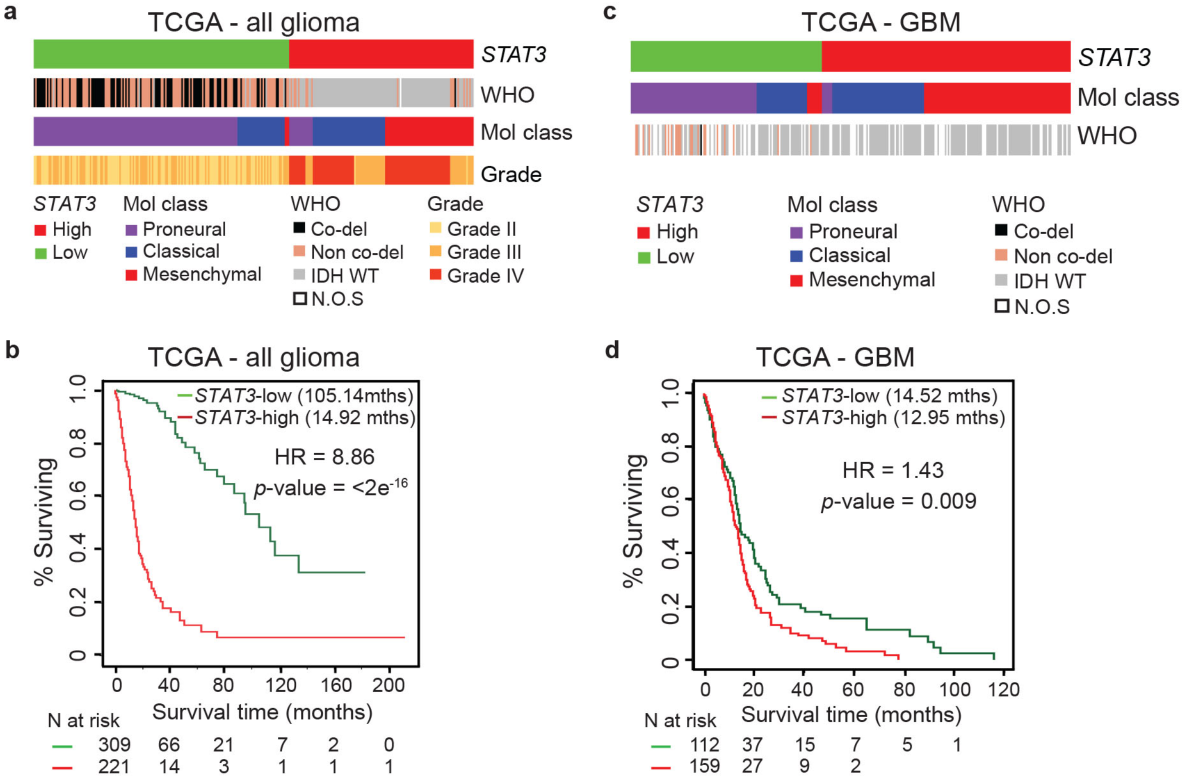

Supplementary Figure 2 NNI-*STAT3* signature validation in TCGA database. In all glioma patients, **a** *STAT3*-high patient cohort was enriched in mesenchymal and classical subtypes with IDH-wild-type status. In contrast, *STAT3*-low tumors comprised mostly low-grade gliomas (LGGs), IDH-mutant (1p/19q co-deleted and non-co-deleted) status and the proneural molecular subtype. **b** NNI-*STAT3* signature stratifies patient survival, with *STAT3*-high patients demonstrating poorer prognosis, 14.92 months compared to 105.14 months in *STAT3*-low cohort. **c** A similar trend was observed in exclusively GBM patients, with **d** *STAT3*-high patients demonstrating poorer survival, 12.95 months compared to 14.52 months in *STAT3*-low cohort.

Supp Fig. 3.

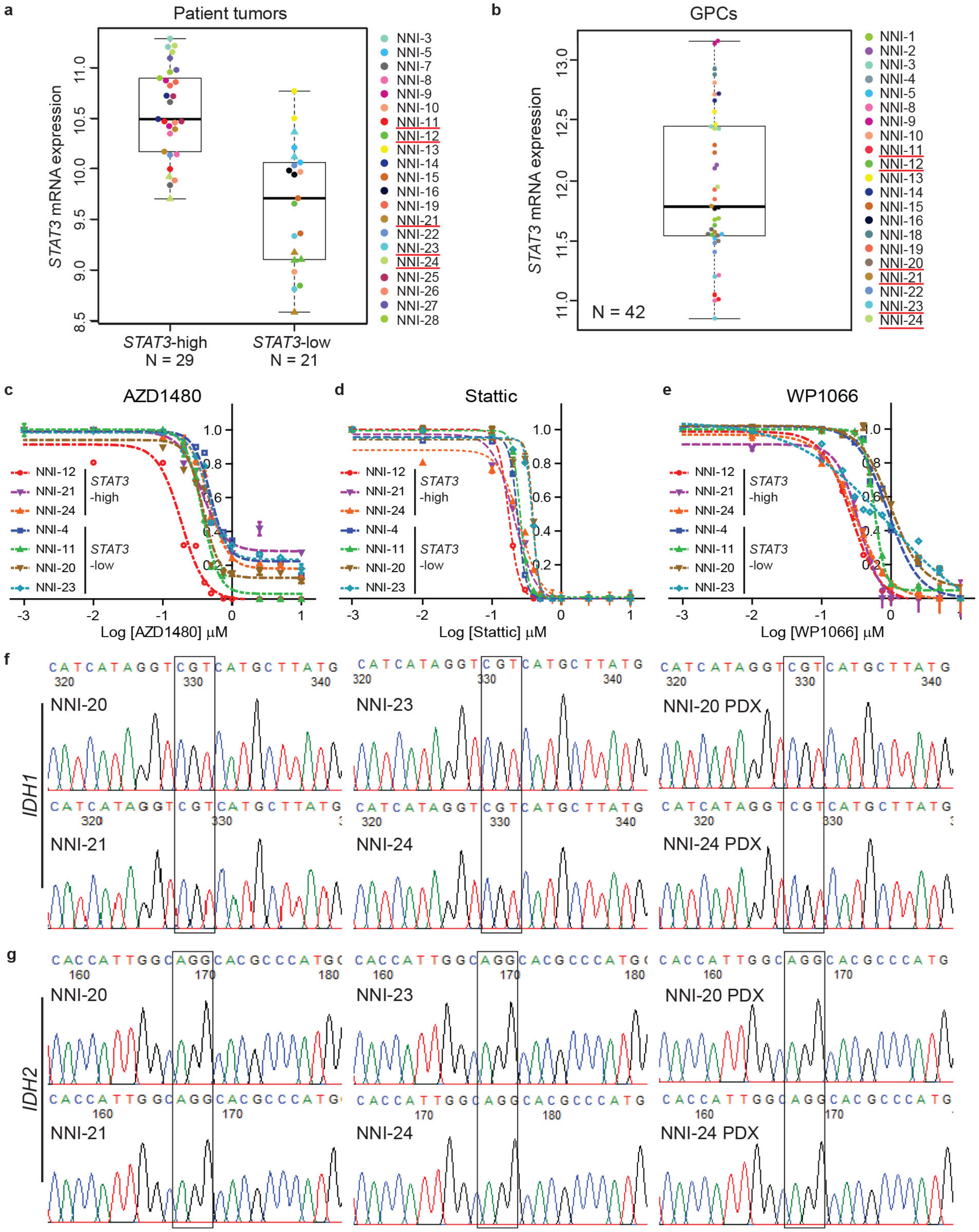
a
Patient tumors
STAT3 mRNA expression
STAT3-high
N = 29
STAT3-low
N = 21
NNI-3
NNI-5
NNI-7
NNI-8
NNI-9
NNI-10
NNI-11
NNI-12
NNI-13
NNI-14
NNI-15
NNI-16
NNI-19
NNI-21
NNI-22
NNI-23
NNI-24
NNI-25
NNI-26
NNI-27
NNI-28
b
GPCs
STAT3 mRNA expression
N = 42
NNI-1
NNI-2
NNI-3
NNI-4
NNI-5
NNI-8
NNI-9
NNI-10
NNI-11
NNI-12
NNI-13
NNI-14
NNI-15
NNI-16
NNI-18
NNI-19
NNI-20
NNI-21
NNI-22
NNI-23
NNI-24
c
AZD1480
Log [AZD1480] μM
d
Stattic
Log [Stattic] μM
e
WP1066
Log [WP1066] μM
NNI-12
NNI-21
NNI-24
STAT3 -high
NNI-4
NNI-11
NNI-20
NNI-23
STAT3 -low
f
IDH1
NNI-20
NNI-23
NNI-20 PDX
NNI-21
NNI-24
NNI-24 PDX
g
IDH2
NNI-20
NNI-23
NNI-20 PDX
NNI-21
NNI-24
NNI-24 PDX

Supplementary Figure 3 NNI patient tumors are IDH-wild-type with variable *STAT3* expression and are stratified by *STAT3* functionally-tuned gene signature. **a** NNI GBM patient tumors, and **b** patient-derived GBM-propagating cells (GPCs) have variable *STAT3* expression and are stratified into *STAT3*-high and –low subtypes. Seven GPCs were treated with **c** AZD1480; **d** Stattic; and **e** WP1066, and their IC_{50} values determined. **f, g** Polymerase chain reaction (PCR)-based sequencing validated that patient cells (NNI-20, 21, 23 and 24) and patient-derived xenografts (NNI-20 and 24) were wild-type for **f** *IDH*1 and **g** *IDH*2.

Supp Fig. 4.

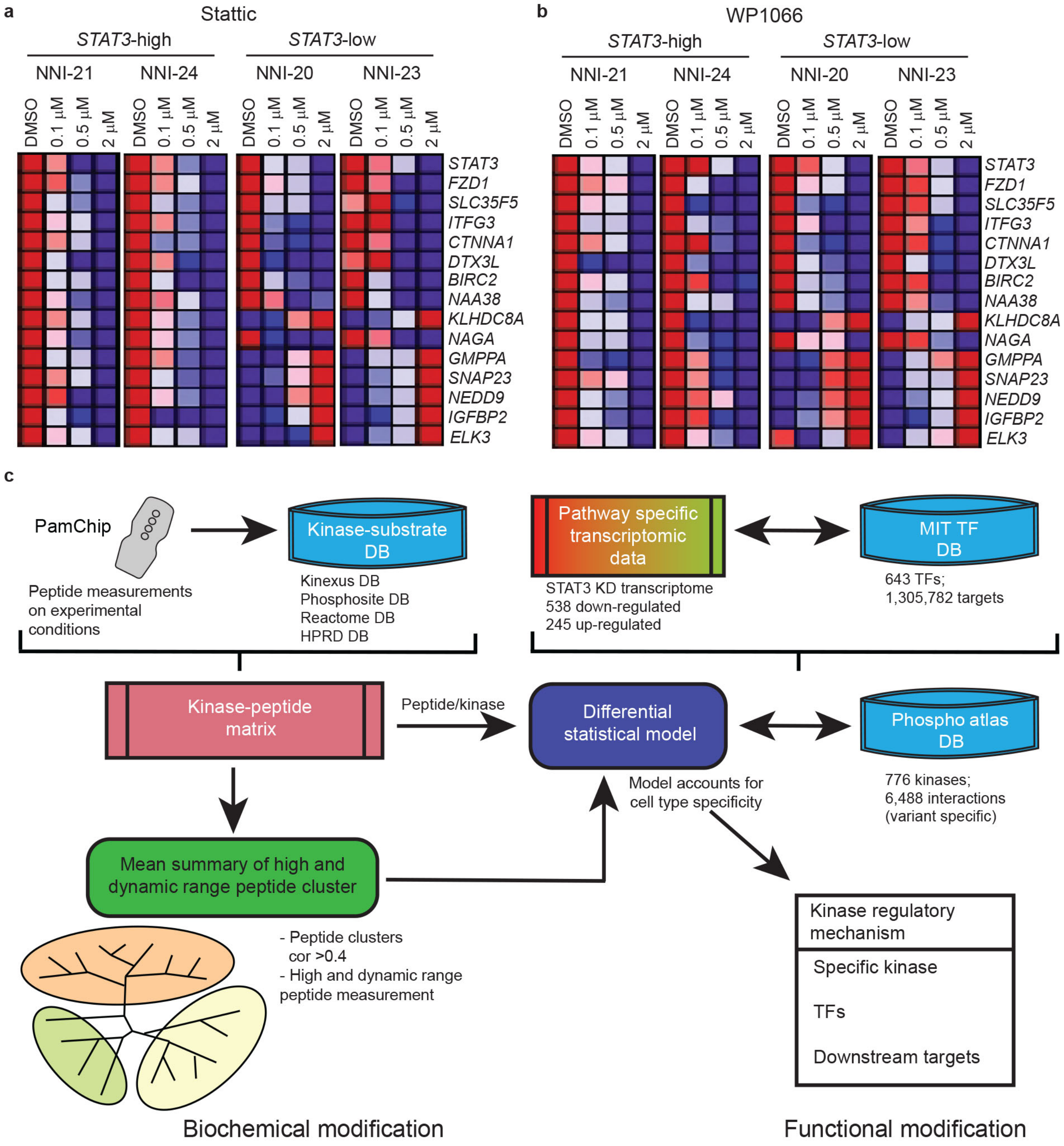
a
Stattic
STAT3-high
STAT3-low
NNI-21
NNI-24
NNI-20
NNI-23
DMSO
0.1 µM
0.5 µM
2 µM
STAT3
FZD1
SLC35F5
ITFG3
CTNNA1
DTX3L
BIRC2
NAA38
KLHDC8A
NAGA
GMPPA
SNAP23
NEDD9
IGFBP2
ELK3
b
WP1066
c
PamChip
Peptide measurements on experimental conditions
Kinase-substrate DB
Kinexus DB
Phosphosite DB
Reactome DB
HPRD DB
Pathway specific transcriptomic data
STAT3 KD transcriptome
538 down-regulated
245 up-regulated
MIT TF DB
643 TFs;
1,305,782 targets
Kinase-peptide matrix
Peptide/kinase
Differential statistical model
Model accounts for cell type specificity
Phospho atlas DB
776 kinases;
6,488 interactions
(variant specific)
Mean summary of high and dynamic range peptide cluster
- Peptide clusters cor >0.4
- High and dynamic range peptide measurement
Kinase regulatory mechanism
Specific kinase
TFs
Downstream targets
Biochemical modification
Functional modification

Supplementary Figure 4 Mechanistic gene candidates identified by NNI-*STAT3* gene signature representing chemoresistance to STAT3 inhibitor. Winnowed gene list across patient tumors identifying candidates uniquely up-regulated in *STAT3*-high subtype. A dose-dependent differential gene expression after STAT3 inhibitor **a** Stattic, and **b** WP1066 treatment in *STAT3*-high and -low cell lines distinguished cooperative genes underlying the *STAT3*-resistant profile. **c** Computational workflow to prioritize kinase candidates with functional/biological phenotypes. The workflow included biochemical modification and functional modules to integrate phosphorylated kinetics measured from PamChip established from *STAT3* knockdown transcriptomic profile. A linear regression model was used to evaluate the kinase activity variable between the cells with treated with AZD1480.

Supp Fig. 5.

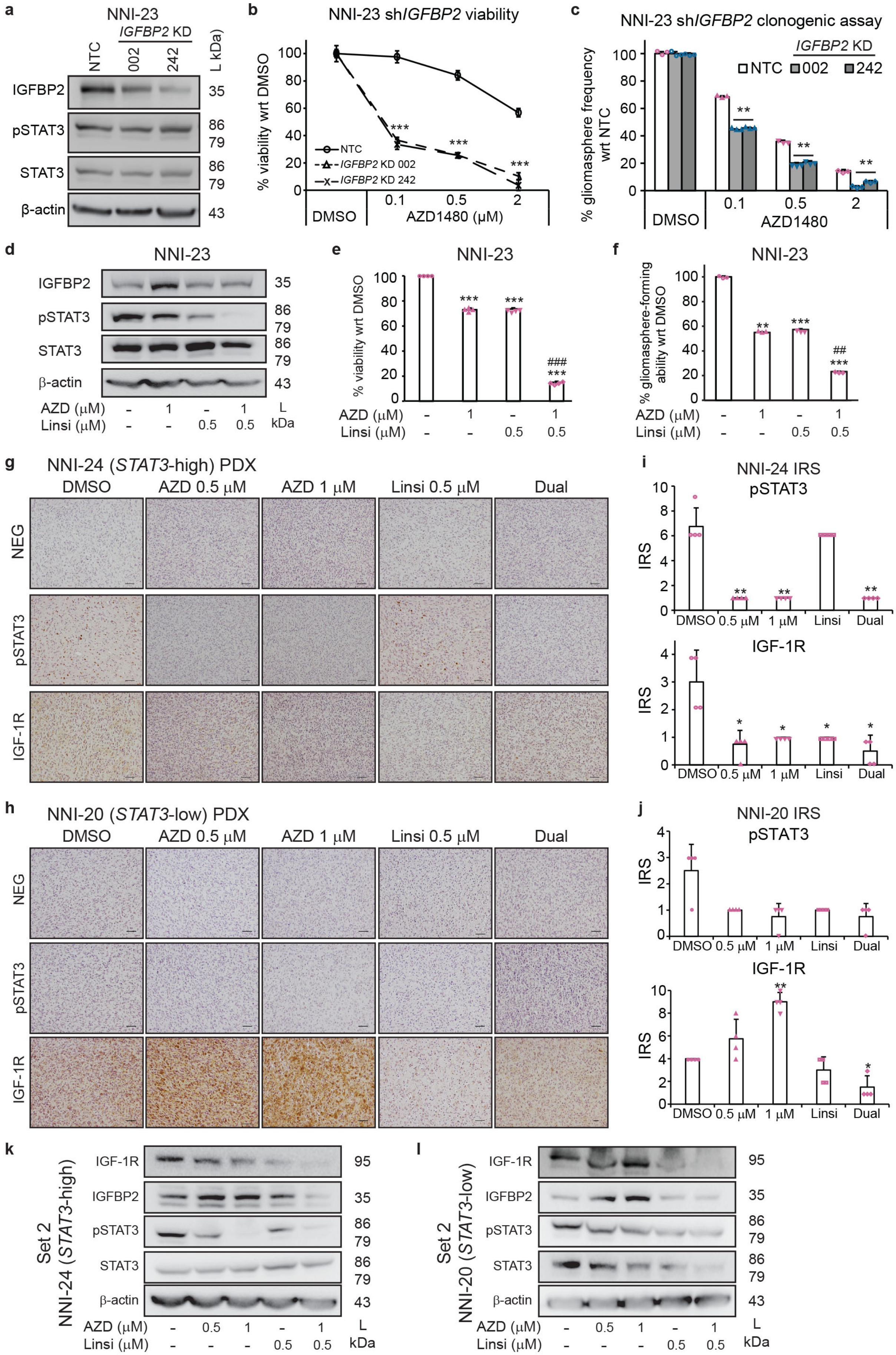

a
NNI-23
IGFBP2 KD
NTC
002
242
L kDa)
IGFBP2
pSTAT3
STAT3
β-actin
b
NNI-23 shIGFBP2 viability
% viability wrt DMSO
NTC
IGFBP2 KD 002
IGFBP2 KD 242
DMSO
AZD1480 (μM)
c
NNI-23 shIGFBP2 clonogenic assay
% gliomasphere frequency wrt NTC
IGFBP2 KD
NTC
002
242
AZD1480
d
NNI-23
AZD (μM)
Linsi (μM)
e
% viability wrt DMSO
f
% gliomasphere-forming ability wrt DMSO
g
NNI-24 (STAT3-high) PDX
DMSO
AZD 0.5 μM
AZD 1 μM
Linsi 0.5 μM
Dual
NEG
pSTAT3
IGF-1R
h
NNI-20 (STAT3-low) PDX
i
NNI-24 IRS
pSTAT3
IRS
Linsi
j
NNI-20 IRS
k
Set 2
NNI-24 (STAT3-high)
l
Set 2
NNI-20 (STAT3-low)

Supplementary Figure 5 Sensitization of *STAT3*-low glioma cells, immunohistochemical (IHC) staining and quantification of pSTAT3 and IGF-1R in patient-derived xenograft (PDX) tumors. **a-c** Depletion of mechanistic gene *IGFBP2*: **a** immunoblot verified *IGFBP2* knockdown. Compared to the non-targeting control (NTC), sh*IGFBP2* clones displayed increased sensitivity to AZD1480, as observed by **b** decreased viability and **c** reduced gliomasphere-forming frequency in an additional *STAT3*-low cell line, NNI-23. **, $p<0.01$; ***, $p<0.001$; KD clones versus NTC. For statistical analysis, two-sided Student's t-test was used. Error bars represent standard deviation of the mean. **d-f** Using a dual drug treatment strategy (AZD1480 against STAT3, and Linsitinib against IGF-1R), NNI-23 demonstrated a reduction of IGF-1R and pSTAT3 as observed in **d** immunoblot analysis, **e** viability and **f** gliomasphere-forming frequency assays. **, $p<0.01$; ***, $p<0.001$; treatment groups versus DMSO control. ##, $p<0.01$; ###, $p<0.001$; dual inhibitors versus individual inhibitor (AZD1480 or Linsitinib). The CI value for the combined drugs, 1 μM AZD1480 and 0.5 μM Linsitinib is 0.23, as calculated using CompuSyn. **g, h** Representative images of PDX tumors stained for pSTAT3 and IGF-1R. **g** PDX-derived tumor from STAT3 inhibitor-treated cells demonstrated a reduction of pSTAT3 expression in NNI-24 (*STAT3*-high). In contrast, **h** NNI-20 (*STAT3*-low) PDX tumors demonstrated a stark increase in IGF-1R expression. Scale bar denotes 50 μm. **i, j** Immunoreactivity scores (IRS) of the PDX tissues were assessed for pSTAT3 and IGF-1R expression. For statistical analysis, two-sided Student's *t* test was used. Error bars represent standard deviation of the mean. **k, l** Second set of immunoblot analysis of PDX tumors, **k** *STAT3*-high, and **l** *STAT3*-low.

Supp Fig. 6.

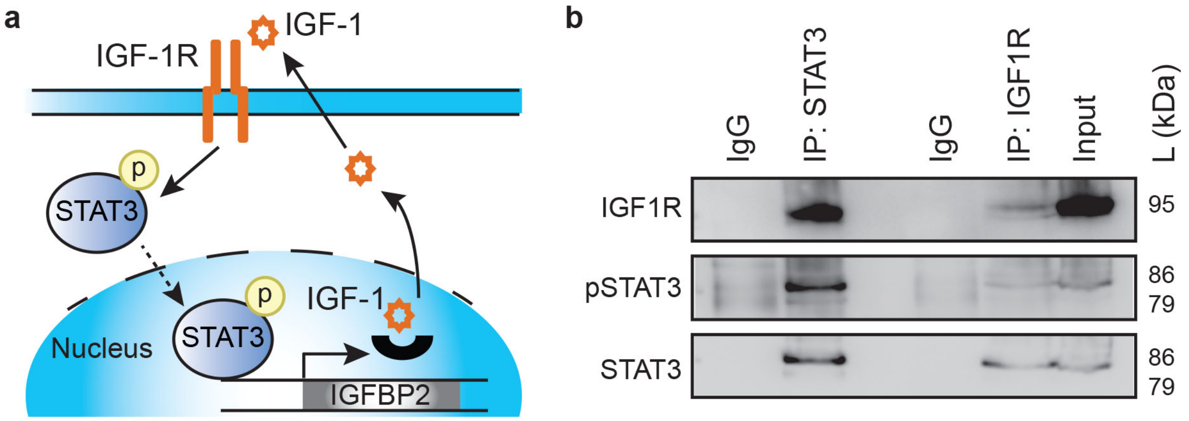

Supplementary Figure 6 Proposed mechanism in *STAT3*-low (chemoresistant) GBM cells. **a** We propose that in *STAT3*-low cells, phosphorylated STAT3 (pSTAT3) activates the transcription of *IGFBP2*, which increases the production of IGF-1 cytokine. Increased production of IGF-1 triggers the activation of the IGF-1R signaling axis, contributing to an, as yet undescribed, feed-forward mechanism. **b** Immunoprecipitation assays demonstrated the physical interaction of IGF-1R with STAT3.

Supp Fig. 7.

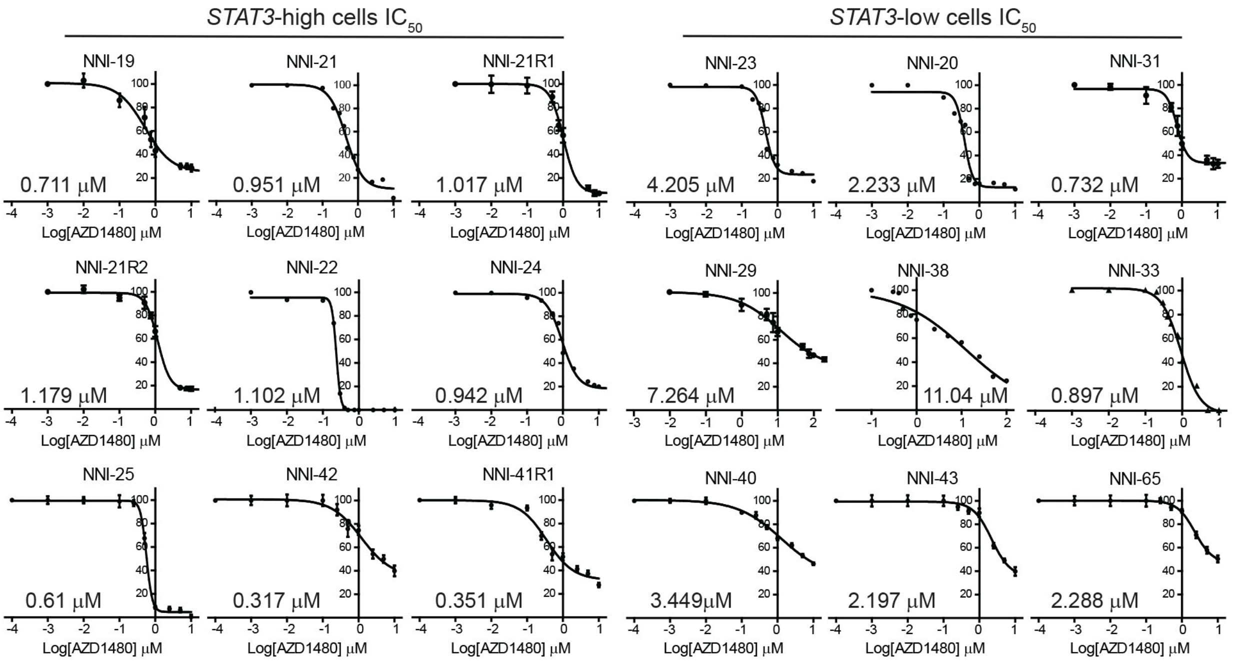

Supplementary Figure 7 IC_{50} values of GBM-propagating cells (GPCs). Dose response curves of **s**tratified *STAT3*-high (N=9) and *STAT3*-low (N=9) cells treated with AZD1480. IC_{50} values were determined from a mean of triplicates, with a 10-point titration curve ranging from 10^{-4} to 10^{2} μM.

Supp Fig. 8.

a

Molecular classes	1st presentation	Recurrence
Mesenchymal (N=11; 35%)	*STAT3*-high (N=7; 64%)	*STAT3*-high (N=6; 86%)
		STAT3-low (N=1; 14%)
	STAT3-low (N=4; 36%)	*STAT3*-high(N=4; 100%)
Classical (N=11; 35%)	*STAT3*-high (N=7; 64%)	*STAT3*-high (N=4; 57%)
		STAT3-low (N=3; 43%)
	STAT3-low (N=4; 36%)	*STAT3*-high (N=3; 75%)
		STAT3-low (N=1; 25%)
Proneural (N=13;30%)	*STAT3*-high (N=4; 31%)	*STAT3*-high (N=3; 75%)
		STAT3-low (N=1; 25%)
	STAT3-low (N=9; 69%)	*STAT3*-high (N=2; 22%)
		STAT3-low (N=7; 78%)

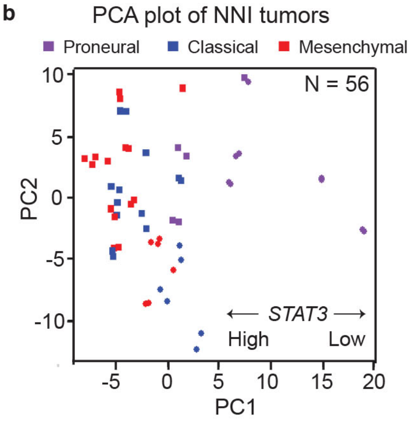

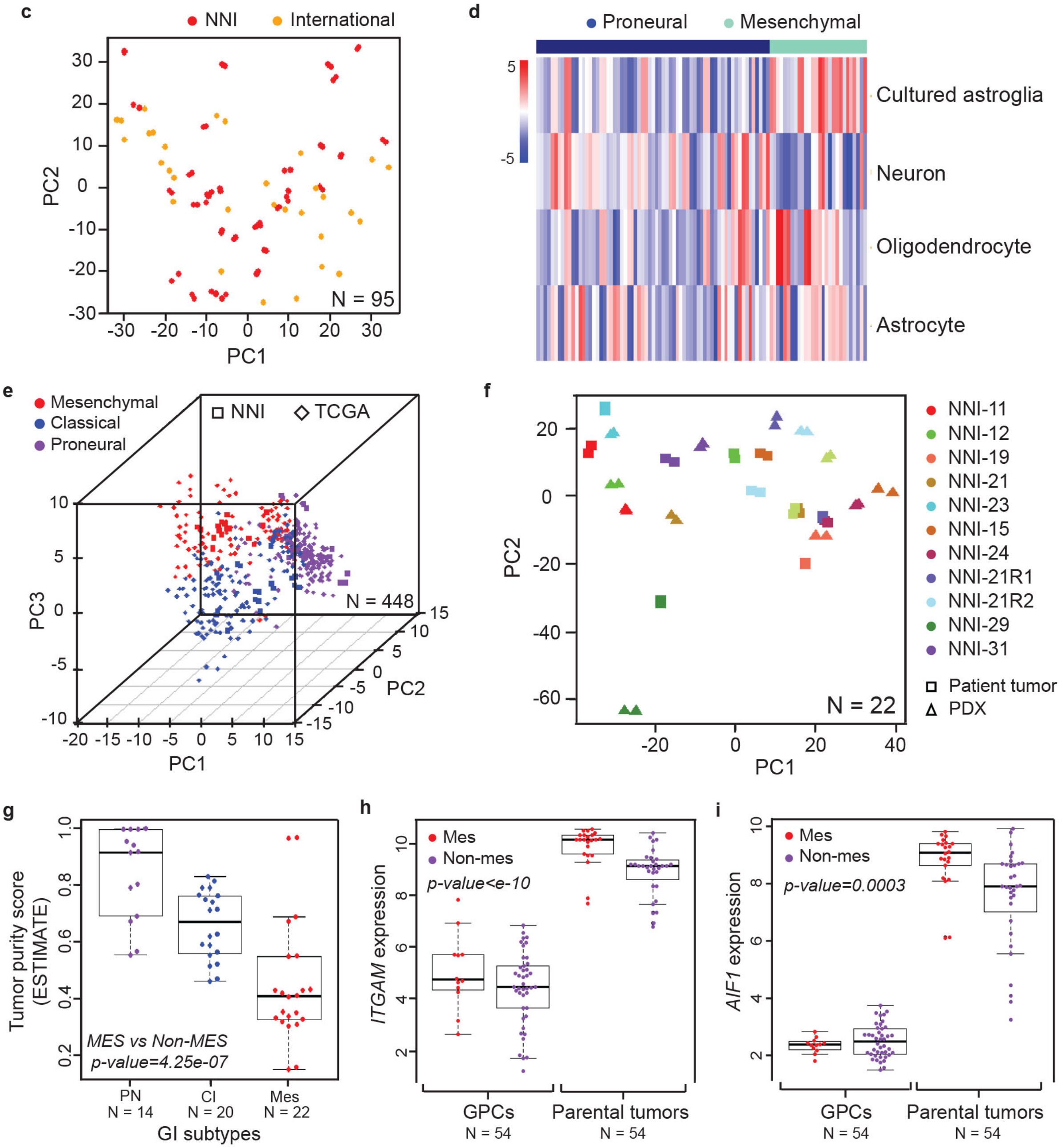

Supplementary Figure 8 Validation of NNI tumors and GPCs with international collections. **a** Using the recurrent GBM tumor databases from Samsung Medical Center and Genentech[1, 2], we evaluated the distribution of matched recurrent patients with subtype switching. Consistent with our earlier data, the responsive cohort remained predominantly as *STAT3*-high mesenchymal tumors upon recurrence. **b** We evaluated our NNI brain tumor resource for the clustering patterns of individual tumors. Noteworthy is *STAT3*-high NNI GBM cells clustered as mesenchymal subtype, while the *STAT3*-low cells clustered as proneural subtype. **c, d** Comparison of NNI patient-derived GBM cells with international collections based on **c** Top most variable probesets, and **d** neural cell types defined by the "Cahoy" signature[3]. **e** Comparison of NNI primary tumors with TCGA tumors based on transcriptomic profile established in glioma-intrinsic signature, i.e. devoid of stromal cell types[4]. Red, mesenchymal; purple, proneural; blue, classical. **f** Comparison of orthotopic PDX tumors (represented as triangle) with matched patient's original tumors (represented as square). **g-i** Tumor purity score association with heterogeneity was evaluated as previously described, and compared between NNI and TCGA specimens[4]. Briefly, the abundance of stromal cell-associated genes, **h** *ITGAM* and **i** *AIF1* was evaluated in GPCs and parental tumors. These values were then computed in ESTIMATE to determine the tumor purity score. NNI primary tumor collection has mean purity of 62%, which is in comparable range with that from TCGA (59%).

Supp Fig. 9.

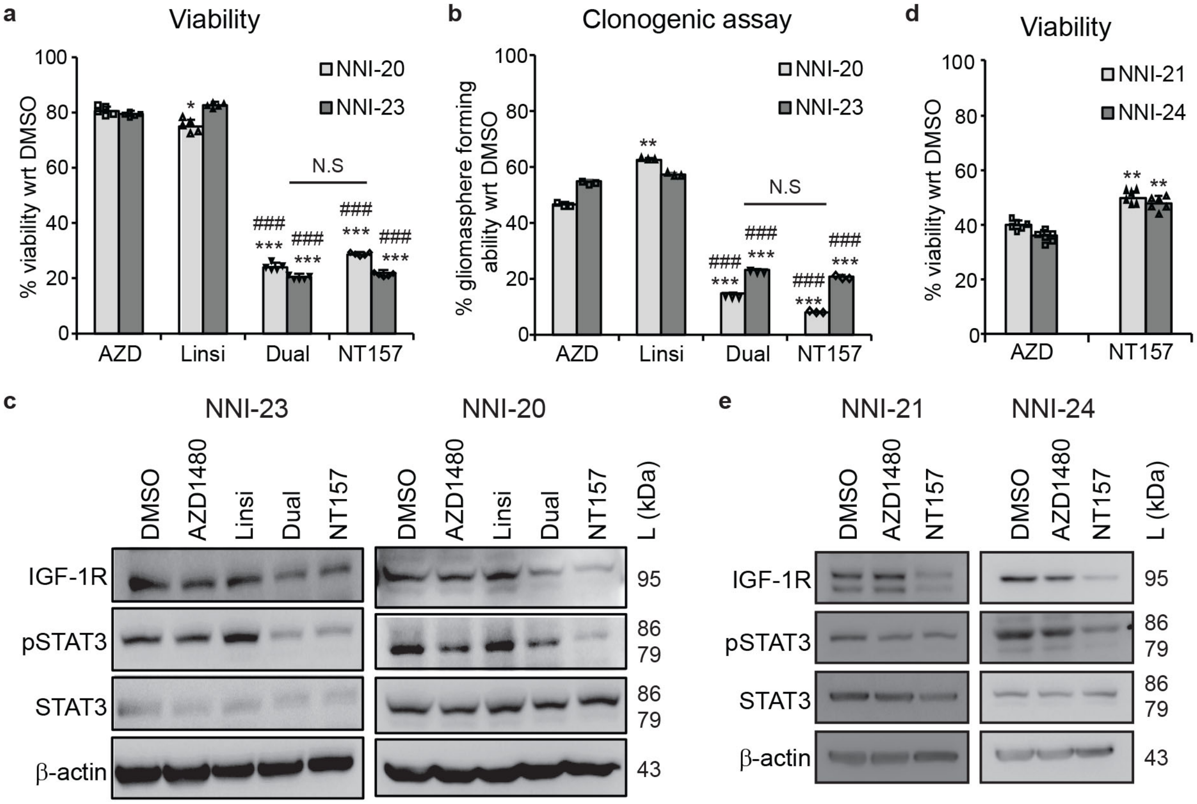

Supplementary Figure 9 Response to NT157, a selective inhibitor targeting IGF-1R and STAT3 signaling pathways. **a** Viability, **b** Clonogenic assay and **c** Immunoblot of *STAT3*-low cells (NNI-20 and NNI-23) to single inhibitor AZD1480 or Linsitinib, a combination of AZD1480 and Linsitinib (Dual), or NT157. There was no significant difference between treatment with NT157 versus a dual inhibitor treatment (AZD1480 and Linsitinib). In contrast, NT157 did not confer additional therapeutic benefit to *STAT3*-high cells (NNI-21 and NNI-24) demonstrated by **d** viability and **e** immunoblot analysis. **, $p<0.01$; ***, $p<0.001$ versus AZD1480; ###, $p<0.001$ versus single inhibitor treatment (AZD1480 or Linsitinib). For statistical analysis, two-sided Student's t-test was used. Error bars represent standard deviation of the mean.

Supplementary Tables

Supplementary Table 1. (a) Contingency tables, (b) Univariate and multivariate analyses.

(a) Contingency tables

Molecular classes:

Gravendeel database

	All Glioma			GBM		
STAT3/Mol classes	*STAT3*-high N (%)	*STAT3*-Low N (%)	Fisher's Exact p-val	*STAT3*-High N (%)	*STAT3*-Low N (%)	Fisher's Exact p-val
Proneural	15 (16%)	79 (84%)	< 2.2e-16	0 (0%)	30 (100%)	< 2.2e-16
Classical	13 (18.8%)	56 (81.2%)		17 (62.96%)	10 (37.04%)	
Mesenchymal	1 (1.5%)	68 (98.5%)		46 (92%)	4 (8%)	

TCGA database

	All Glioma			GBM		
STAT3/Mol classes	*STAT3*-High N (%)	*STAT3*-Low N (%)	Fisher's Exact p-val	*STAT3*-High N (%)	*STAT3*-Low N (%)	Fisher's Exact p-val
Proneural	28 (10.1%)	248 (89.9%)	< 2.2e-16	7 (7.955%)	81 (92.045%)	< 2.2e-16
Classical	87 (60.8%)	56 (39.2%)		59 (64.13%)	33 (35.87%)	
Mesenchymal	107 (93.9%)	7 (6.1%)		94 (91.262%)	9 (8.737%)	

W.H.O classes:

Gravendeel database

	All Glioma			GBM		
STAT3/W.H.O	*STAT3*-High N (%)	*STAT3*-Low N (%)	Fisher's Exact p-val	*STAT3*-High N (%)	*STAT3*-Low N (%)	Fisher's Exact p-val
IDH1 mut CDL	2 (8.7%)	21 (91.3%)	< 7.22e-11	0 (0%)	3 (100%)	0.012
IDH1 mut NCDL	19 (44.2%)	24 (55.9%)		10 (43.478%)	13 (56.522%)	
IDH1 WT	93 (76.9%)	28 (23.1%)		40 (67.797%)	19 (32.203%)	

TCGA database

	All Glioma			GBM		
STAT3/W.H.O	*STAT3*-High N (%)	*STAT3*-Low N (%)	Fisher's Exact p-val	*STAT3*-High N (%)	*STAT3*-Low N (%)	Fisher's Exact p-val
IDH mut CDL	3 (2.1%)	141 (97.9%)	< 2.2e-16	0 (0%)	1 (100%)	7.3e-10
IDH mut NCDL	29 (16%)	152 (84%)		0 (0%)	20 (100%)	
IDH WT	179 (91.8%)	16 (8.2%)		120 (67.039%)	59 (32.961%)	

Grade:

Gravendeel database

	All Glioma		
STAT3/Grade	*STAT3*-High N (%)	*STAT3*-Low N (%)	Fisher's Exact p-val
Grade II	1 (3.448%)	28 (96.552%)	< 2.2e-16
Grade III	21 (28.378%)	53 (71.622%)	
Grade IV	106 (78.519%)	29 (21.481%)	

TCGA database

	All Glioma		
STAT3/Grade	*STAT3*-High N (%)	*STAT3*-Low N (%)	Fisher's Exact p-val
Grade II	1 (1%)	99 (99%)	< 2.2e-16
Grade III	29 (28.155%)	74 (71.845%)	
Grade IV	145 (98.639%)	2 (1.361%)	

(b) Univariate and multivariate analyses

Gravendeel database

All Glioma

<table>
<tr><td>Covariates</td><td colspan="3">Univariate analysis</td><td colspan="3">Multivariate analysis</td></tr>
<tr><td></td><td>HR (95% CI)</td><td>SE</td><td>Pr(>|z|)*</td><td>HR (95% CI)</td><td>SE</td><td>Pr(>|z|)*</td></tr>
<tr><td>STAT3-High</td><td>4.587 (3.327-6.324)</td><td>0.164</td><td><2e-16</td><td>3.366 (1.978-5.729)</td><td>0.272</td><td>7.78e-06</td></tr>
<tr><td>W.H.O IDH mut NCDL</td><td>1.932 (1.11-3.36)</td><td>0.283</td><td>0.02</td><td>2.1 (1.169-3.776)</td><td>0.299</td><td>0.013</td></tr>
<tr><td>W.H.O IDH WT</td><td>2.68 (1.628-3.36)</td><td>0.254</td><td>1.00e-04</td><td>1.864 (1.05-3.307)</td><td>0.293</td><td>0.033</td></tr>
<tr><td>GI Classical</td><td>2.2 (1.559-3.105)</td><td>0.176</td><td>7.29e-06</td><td>0.891 (0.564-1.407)</td><td>0.233</td><td>0.62</td></tr>
<tr><td>GI Mesenchymal</td><td>2.691 (1.906-3.801)</td><td>0.176</td><td>1.90e-08</td><td>0.845 (0.508-1.407)</td><td>0.26</td><td>0.518</td></tr>
<tr><td>Age</td><td>1.045 (1.034-1.057)</td><td>0.006</td><td>8.49e-15</td><td>1.041 (1.027-1.055)</td><td>0.007</td><td>2.98e-9</td></tr>
</table>

GBM

<table>
<tr><td>Covariates</td><td colspan="3">Univariate analysis</td><td colspan="3">Multivariate analysis</td></tr>
<tr><td></td><td>HR (95% CI)</td><td>SE</td><td>Pr(>|z|)*</td><td>HR (95% CI)</td><td>SE</td><td>Pr(>|z|)*</td></tr>
<tr><td>STAT3-High</td><td>1.765 (1.156-2.694)</td><td>0.216</td><td>0.0085</td><td>1.343 (0.811-2.226)</td><td>0.258</td><td>0.252</td></tr>
<tr><td>W.H.O IDH mut NCDL</td><td colspan="6">-</td></tr>
<tr><td>W.H.O IDH WT</td><td>1.801 (1.048-3.094)</td><td>0.276</td><td>0.0332</td><td>1.211 (0.676-2.171)</td><td>0.298</td><td>0.52</td></tr>
<tr><td>GI Classical</td><td>1.556 (0.881-2.749)</td><td>0.29</td><td>0.127</td><td colspan="3" rowspan="2">-</td></tr>
<tr><td>GI Mesenchymal</td><td>1.35 (0.826-2.208)</td><td>0.251</td><td>0.231</td></tr>
<tr><td>Age</td><td>1.036 (1.018-1.053)</td><td>0.009</td><td>4.38e-05</td><td>1.03 (1.01-1.05)</td><td>0.01</td><td>0.002</td></tr>
</table>

TCGA database

All Glioma

Covariates	Univariate analysis			Multivariate analysis		
	HR (95% CI)	SE	Pr(>|z|)*	HR (95% CI)	SE	Pr(>|z|)*
STAT3-High	10.687 (7.168-15.93)	0.204	<2e-16	2.568 (1.302-5.067)	0.347	0.007
W.H.O IDH mut NCDL	1.61 (0.808-3.205)	0.351	0.176	1.679 (0.809-3.488)	0.373	0.164
W.H.O IDH WT	19.346 (10.393-36.01)	0.317	<2e-16	4.215 (1.695-10.486)	0.465	0.002
GI Classical	5.937 (3.832-9.196)	0.223	1.50e-15	1.507 (0.829-2.741)	0.305	0.178
GI Mesenchymal	8.057 (5.261-12.337)	0.217	<2e-16	1.553 (0.847-2.85)	0.31	0.155
Age	1.076 (1.063-1.09)	0.006	<2e-16	1.05 (1.034-1.066)	0.008	7.37e-10

GBM

Covariates	Univariate analysis			Multivariate analysis		
	HR (95% CI)	SE	Pr(>|z|)*	HR (95% CI)	SE	Pr(>|z|)*
STAT3-High	1.457 (1.099-1.932)	0.144	0.009	1.063 (0.709-1.595)	0.207	0.767
W.H.O IDH mut NCDL	-					
W.H.O IDH WT	3.198 (1.7-6.015)	0.322	0.0003	2.036 (0.971-4.268)	0.378	0.06
GI Classical	1.51 (1.06-2.152)	0.181	0.022	0.698 (0.429-1.136)	0.249	0.148
GI Mesenchymal	1.534 (1.083-2.172)	0.178	0.016	0.884 (0.527-1.483)	0.264	0.64
Age	1.036 (1.024-1.047)	0.006	2.31e-10	1.04 (1.025-1.055)	0.008	1.85e-07

**STAT3*-low patient cohort was considered a reference category to estimate the coefficient in Cox regression model; W.H.O-IDH Mut-CDL (Codeleted for chromosome 1p/19q) patients were treated as the reference to estimate the coefficient in Cox regression model; GI (Glioma Intrinsic) - Proneural patients were treated as the reference to estimate the coefficient in Cox regression model.

CI = confidence interval; HR = Hazard ratio; Mut = Mutation; NCDL = non-codeleted for chromosome 1p/19q; Pr(<|z|) = two-sided Wald test p-value; SE = standard error of coefficient.

Supplementary Table 2. Kaplan-Meier statistics.

GBM Cell Line	Treatment	Median Survival (Days)	Hazard ratio	Logrank p-val versus DMSO
NNI-24 *STAT3-high*	DMSO (N=8)	134	1.00	-
	AZD1480, 0.5 μM (N=8)	171	25.17	***, 0.0001
	AZD1480, 1 μM (N=8)	181	25.17	***, <0.0001
	Linsitinib, 0.5 μM (N=8)	161	6.83	**, 0.0044
	Dual (N=8)	192	20.07	***, <0.0001
NNI-20 *STAT3*-Low	DMSO (N=8)	135	1.00	-
	AZD1480, 0.5 μM (N=8)	143	1.14	NS, 0.8118
	AZD1480, 1 μM (N=8)	154	3.53	*, 0.041
	Linsitinib, 0.5 μM (N=8)	137	0.90	NS, 0.8373
	Dual (N=7)	200	25.17	***, <0.0001

Supplementary Table 3. List of protein tyrosine kinases up/down-regulated in *STAT3*-high and –low GPCs upon treatment with AZD1480.

***STAT3*-High**

Kinases	Log_2 Fold change	p-val	FDR
ERK1	-0.130003	0.000855	0.023359
ERK2	-0.130003	0.000855	0.023359
RAF1	-0.130003	0.000855	0.023359
BLK	0.116378	0.003183	0.065257
ROR2	-0.132880	0.009185	0.110608
JAK3	-0.072164	0.009442	0.110608
JAK3~b	-0.072164	0.009442	0.110608
Ret	-0.083986	0.014765	0.151341
Lyn	0.060460	0.020579	0.172812
Ron	0.071592	0.022478	0.172812
EphA4	-0.164597	0.023182	0.172812
EphA10	-0.075429	0.035620	0.240748
Syk	-0.140010	0.038167	0.240748
Srm	0.065889	0.049258	0.288510
PKA[alpha]	0.046967	0.096575	0.527944

***STAT3*-Low**

Kinases	Log_2 Fold change	p-val	FDR
Srm	0.5972285	0.0215359	0.1818153
DDR2	0.5897400	0.0304162	0.1818153
DYRK1B	0.5807676	0.0311966	0.1818153
CTK	-0.4509096	0.0388479	0.1818153
Kit	0.6433738	0.0400803	0.1818153
IGF1R	0.3345388	0.0421874	0.1818153
ITK	0.4015083	0.0451334	0.1818153
Mer	0.6474189	0.0468634	0.1818153
ZAP70	-0.3271584	0.0497777	0.1818153
FGFR4	0.3781613	0.0500557	0.1818153
FAK	0.6526299	0.0502942	0.1818153
EGFR	0.3924259	0.0503176	0.1818153
ASK/MAP3K5	0.4158566	0.0511232	0.1818153
MAP2K7	0.4158566	0.0511232	0.1818153
MEKK6/MAP3K6	0.4158566	0.0511232	0.1818153
SEK1/MAP2K4	0.4158566	0.0511232	0.1818153
Brk	-0.3391364	0.0533396	0.1818153
Tyro3/Sky	0.3446778	0.0536684	0.1818153
ALK	-0.4122881	0.0566978	0.1818153
FGFR1	0.4025010	0.0568583	0.1818153
Ret	0.4072459	0.0573280	0.1818153
DDR1	0.3654692	0.0586528	0.1818153
InSR	-0.3360124	0.0593668	0.1818153
Yes	-0.3953469	0.0627906	0.1818153
Src	-0.2713709	0.0631483	0.1818153
ERK1	0.3986351	0.0672459	0.1818153
RK2	0.3986351	0.0672459	0.1818153
RAF1	0.3986351	0.0672459	0.1818153
EphA10	0.3856443	0.0687550	0.1818153
Ron	0.3677418	0.0690711	0.1818153
Fes	-0.3333383	0.0700469	0.1818153
PYK2	0.7014802	0.0709523	0.1818153
TXK	0.7321577	0.0775809	0.1927768
CSK	0.7554339	0.0845210	0.2038449
JAK3	-0.3007821	0.0922822	0.2101983
JAK3~b	-0.3007821	0.0922822	0.2101983
MEK2/MAP2K2	0.4172901	0.0963204	0.2134668

Supplementary Table 4. List of top synergistic compounds able to reverse *STAT3*-high GBM disease signature (temozolomide as reference compound).

Drug	LINCS ID	*STAT3*-high GBM Signature Discordance	TMZ Orthogonality
Ruxolitinib	LSM-1139	1	0.011236
LCQ-908	LSM-45255	0.5626	0
Procaine	LSM-5396	0.5	0
Chlorthalidone	LSM-1417	0.4375	0.011236
Nilotinib	LSM-1099	0.4375	0
Alectinib	LSM-1202	0.375	0
BRD-K68548958	LSM-43281	0.375	0.003745
Indapamide	LSM-1936	0.375	0.011236
Taltirelin	LSM-45418	0.375	0
Timolol	LSM-15524	0.375	0
Anamorelin	LSM-45737	0.3125	0
AZD1480	LSM-1140<br>LSM-45764	0.3125	0

Supplementary References

1. Kim J*, et al.* Spatiotemporal Evolution of the Primary Glioblastoma Genome. *Cancer Cell* **28**, 318-328 (2015).

2. Phillips HS*, et al.* Molecular subclasses of high-grade glioma predict prognosis, delineate a pattern of disease progression, and resemble stages in neurogenesis. *Cancer Cell* **9**, 157-173 (2006).

3. Cahoy JD*, et al.* A transcriptome database for astrocytes, neurons, and oligodendrocytes: a new resource for understanding brain development and function. *J Neurosci* **28**, 264-278 (2008).

4. Wang Q*, et al.* Tumor Evolution of Glioma-Intrinsic Gene Expression Subtypes Associates with Immunological Changes in the Microenvironment. *Cancer Cell* **33**, 152 (2018).
