# Supplementary material for: A STAT3-based gene signature stratifies glioma patients for targeted therapy
Source: Nat Commun. 2019 Aug 9;10:3601. doi: 10.1038/s41467-019-11614-x (PMC6689009; doi:10.1038/s41467-019-11614-x)
Supplement: Supplementary file 3 — Description of Additional Supplementary Files [file 41467_2019_11614_MOESM3_ESM.pdf]

Description of Additional Supplementary Files

File Name: Supplementary Data 1
Description: List of genes comprising the STAT3 functionally-tuned gene signature.

File Name: Supplementary Data 2
Description: Gene Set Enrichment Analysis (GSEA) ranked gene list.

File Name: Supplementary Data 3
Description: Winnowed list of genes contributing to chemoresistance.
